# Enhanced Chromium (VI) Removal by Micron-Scale Zero-Valent Iron Pretreated with Aluminum Chloride under Aerobic Conditions

**DOI:** 10.3390/molecules29102350

**Published:** 2024-05-16

**Authors:** Xuejun Long, Rui Li, Jun Wan, Zhenxing Zhong, Yuxuan Ye, Jiazhi Yang, Jun Luo, Jin Xia, Yaomeng Liu

**Affiliations:** 1 School of Environmental Engineering, Wuhan Textile University, Wuhan 430200, China; 2 State Key Laboratory of New Textile Materials and Advanced Processing Technologies, Wuhan Textile University, Wuhan 430200, China; 3 Engineering Research Center of Ministry of Education for Clean Production of Textile Dyeing and Printing, Wuhan Textile University, Wuhan 430200, China

**Keywords:** adsorption, reduction, Cr(VI) removal, pre-corrosion, zero-valent iron

## Abstract

Micron-scale zero-valent iron (ZVI)-based material has been applied for hexavalent chromium (Cr(VI)) decontamination in wastewater treatment and groundwater remediation, but the passivation problem has limited its field application. In this study, we combined aluminum chloride solution with ZVI (pcZVI-AlCl_3_) to enhance Cr(VI) removal behavior under aerobic conditions. The optimal pre-corrosion conditions were found to be 2.5 g/L ZVI, 0.5 mM AlCl_3_, and a 4 h preconditioning period. Different kinds of techniques were applied to detect the properties of preconditioned ZVI and corrosion products. The ^57^Fe Mössbauer spectra showed that proportions of ZVI, Fe_3_O_4_, and FeOOH in pcZVI-AlCl_3_ were 49.22%, 34.03%, and 16.76%, respectively. The formation of Al(OH)_3_ in the corrosion products improved its pH_pzc_ (point of zero charge) for Cr(VI) adsorption. Continuous-flow experiments showed its great potential for Cr(VI) removal in field applications. The ZVI and corrosion products showed a synergistic effect in enhancing electron transfer for Cr(VI) removal. The mechanisms underlying Cr(VI) removal by pcZVI-AlCl_3_ included adsorption, reduction, and precipitation, and the contribution of adsorption was less. This work provides a new strategy for ZVI pre-corrosion to improve its longevity and enhance Cr(VI) removal.

## 1. Introduction

Chromium (Cr) has been used in the textile, dyeing, plating, and leather industries and generates wastewater containing Cr species that can have toxic effects on human beings, such as lung cancer. Generally, Cr exists in both water and soil in the form of toxic hexavalent chromium (Cr(VI)) and less toxic trivalent chromium (Cr(III)). In addition, Cr(VI) is more mobile in the water environment [1,2,3]. To ensure health goals, the U.S. Environmental Protection Agency requested a concentration below 0.1 mg/L for Cr(VI) in drinking water [4], and this requirement is 0.05 mg/L in the national standard GB 5749-2022 of China [5].

In the past decades, some techniques have been applied in Cr(VI) decontamination in wastewater treatment and groundwater remediation, including adsorption [6], membrane process [7], photocatalytic reduction [8], electrochemical methods [9,10], and biological treatment [11]. Compared with these methods, the zero-valent iron-based technologies have garnered lots of attention because of their low cost, environmental friendliness, easy operation, and efficient performance [12,13]. However, a major challenge with ZVI is the passivation problem, where generated iron hydroxides cover its surface, significantly reducing its reactivity and longevity under different conditions [14]. This limitation has hindered the widespread application of ZVI in the field.

To enhance the utilization efficiency and sustained performance of ZVI, numerous countermeasures have been devised by researchers. These include sulfidation of ZVI [15], ligand-modified ZVI [16], ZVI–Fe_3_O_4_ composites [12], iron–carbon composites [17], iron/aluminum bimetals [18], ZVI@Fe_2_O_3_ [19], and ZVI@Mg(OH)_2_ [20]. The modifications to the Fe^0^ surface primarily aim to enhance its durability and contaminant removal capacity through three key strategies: (1) establishing a core-shell structure to mitigate the passivation reaction, (2) constructing a galvanic cell to boost electrochemical corrosion, and (3) strengthening the electron transfer of ZVI. Despite these efforts, it remains challenging to further improve ZVI’s longevity and reactivity through a cost-effective pretreatment approach for reducing chromate (Cr(VI)) content in wastewater treatment and environmental remediation applications.

Recently, the pre-corrosion of micron-scale ZVI with strong oxidants such as H_2_O_2_ or NaClO has been found to enhance its corrosion extent and successfully promote the removal of oxyanions (e.g., selenate and phosphate) through adsorption and reduction [21,22]. Alternatively, dissolved oxygen (DO) is a cost-effective oxidant for ZVI preconditioning [23]. Furthermore, studies have shown that the pre-corrosion time and the presence of salts like NaCl in the solution significantly influence the morphology and composition of corrosion products, thereby affecting arsenate, selenate, and phosphate removal performance [24,25]. Additionally, chloride (Cl^−^) can trigger pitting corrosion in the solution to enhance the contaminant removal [26].

While the impact of anions on pre-corrosion micron-scale zero-valent iron and the subsequent contaminant removal has been extensively studied [25], there is limited knowledge about the potential role of multivalent cations, such as Al^3+^. These cations could potentially incorporate into the formation of corrosion products and promote Cr(VI) removal. Notably, trivalent aluminum species (e.g., Al_2_O_3_, AlOOH, and Al(OH)_3_) with a high pH_pzc_ (point of zero charge) are favorable adsorbents for oxyanions, including chromate (Cr(VI)) [27,28]. Given these findings, it is anticipated that the combination of Al^3+^ and Cl^−^ in a solution will prevent ZVI passivation by changing the local pH. Thus, pre-corroded ZVI by AlCl_3_ solution could potentially exhibit improved longevity and enhanced Cr(VI) sequestration capabilities.

Therefore, the aims of this study were: (1) to search for an adequate AlCl_3_ dose applied to ZVI to obtain a maximum Cr(VI) removal, (2) to elucidate the individual role of Al^3+^ and Cl^−^ to iron corrosion, (3) to assess the longevity of the pre-corroded ZVI, and (4) to explore the Cr(VI) removal performance and underlying mechanisms of pcZVI-AlCl_3_. This research is expected to facilitate our understanding of ZVI pretreatment with AlCl_3_ solution towards Cr(VI) removal in field applications. The meanings of the symbols used in the research are presented in Table 1.

## 2. Results and Discussion

### 2.1. ZVI Pre-Corrosion Optimization and Characterization

#### 2.1.1. ZVI Preconditioning Process Optimization via Batch Experiments

The effect of ZVI dosage on pre-corrosion for Cr(VI) decontamination was first explored, as shown in Figure S2a. The Cr(VI) removal efficiency increased from 19.70% to approximately 100%, with ZVI dosage increasing from 0.5 g/L to 2.5 g/L. Thus, 2.5 g/L of ZVI was used in the following optimization procedures.

The effect of ZVI pre-corrosion time is presented in Figure S2b. Herein, the pre-corrosion time affects the ZVI corrosion extent, as well as the composition of the corrosion products, which probably strengthens or impedes Cr(VI) removal. The removal efficiency increased from 58.34% to 97.3% when the pretreated time increased from 1 to 4 h. With the pretreated time further raised from 4 to 12 h, the Cr(VI) removal efficiency exhibited a slightly declining trend. Therefore, the pre-corrosion time of 4 h was applied in further experiments.

The effect of initial AlCl_3_ concentration on ZVI pre-corrosion for Cr(VI) removal is exhibited in Figure S2c. The AlCl_3_ dose affected the ZVI pre-corrosion by generating an acidic environment, and the Cr(VI) removal efficiencies under all conditions were above 90%. The AlCl_3_ concentration of 0.5 mM was selected for further experiments due to the relatively high removal efficiency (>97%) and relatively low dosage.

The dissolved oxygen served as the oxidant in ZVI pre-corrosion, and Figure S2d presents its effect on Cr(VI) removal. Unexpectedly, the Cr(VI) removal efficiencies were quite stable (ranging from 89.19% to 93.87%) when the initial DO concentration varied from 0.5 to 10 mg/L. This result suggests that DO concentration had less influence on ZVI pre-corrosion towards Cr(VI) removal.

#### 2.1.2. Corrosion Product Characterization

**Figure 1:**
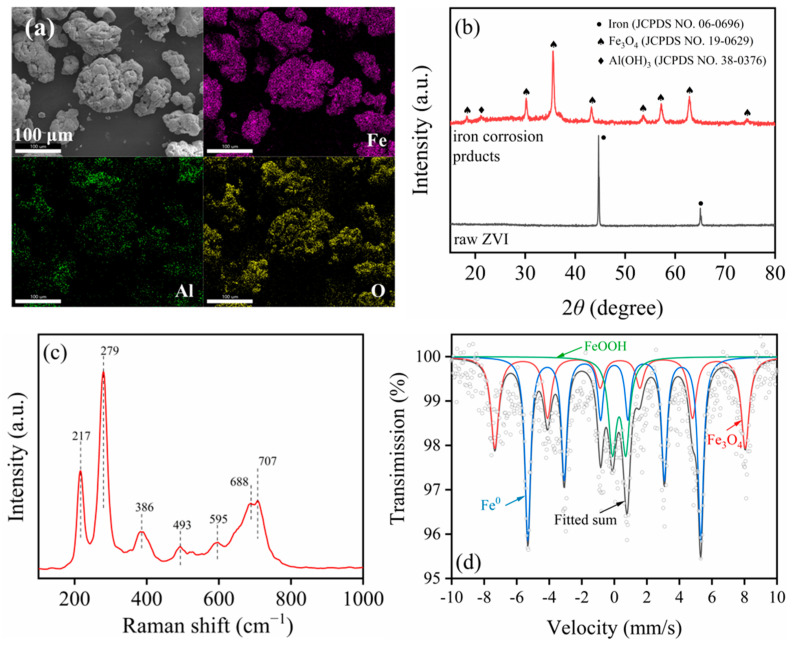
SEM image and elemental mappings (**a**) and ^57^Fe Mössbauer spectra (**d**) of micron-scale ZVI particles after AlCl_3_ solution pretreatment, and XRD patterns (**b**) and Raman spectra (**c**) of separated corrosion products.

The SEM image of the pretreated ZVI is shown in Figure 1a, and the size of these particles was around or lower than 100 μm. Additionally, the elemental mapping results indicated the Fe, Al, and O elements existed on the surface of ZVI after pre-corrosion.

Figure 1b exhibits the XRD patterns of raw ZVI and corrosion products after pre-corrosion. The two typical peaks of ZVI at 44.6° and 65.1° were in accordance with iron (JCPDS No. 06-0696). For the corrosion products, the main peaks at 18.3°, 30.1°, 35.5°, 43.1°, 53.6°, 57.1°, 62.8°, and 74.2° were consistent with Fe_3_O_4_ (JCPDS No. 19-0629). The diffraction peak at 21.1° was ascribed to Al(OH)_3_ (JCPDS No. 38-0376). Therefore, the corrosion products on the ZVI surface were a mixture of iron oxides and Al(OH)_3_.

The Raman spectrum of the above corrosion products is shown in Figure 1c. The bands at 217 cm^−1^ and 279 cm^−1^ were in agreement with magnetite (Fe_3_O_4_) [29]. The bands at 389 cm^−1^ and 688 cm^−1^ were in accordance with goethite (α-FeOOH), and the band at 493 cm^−1^ was due to the akaganeite (β-FeOOH) [30]. The bands at 595 cm^−1^ and 707 cm^−1^ originated from wustite (FeO) and Al(OH)_3_, respectively [31,32]. These results were similar to XRD characterization, and the FeOOH and FeO species were not found in XRD patterns due to their low concentrations or amorphous properties.

To further investigate the iron species and proportions in the corrosion, the ^57^Fe Mössbauer spectra were performed, as shown in Figure 1d and Table S1. The solid phase of iron species in pcZCI mainly contained ZVI, Fe_3_O_4_, and FeOOH, and their content was 49.22%, 34.03%, and 16.76%, respectively. After thorough analysis, it was determined that the corrosion products predominantly comprised iron oxides and Al(OH)_3_, with Fe_3_O_4_ emerging as the primary constituent.

### 2.2. The Role of A^l3+^ and Cl^−^ in ZVI Pre-Corrosion

#### 2.2.1. Cr(VI) Removal by ZVI Pretreatment with Different Solutions

**Figure 2:**
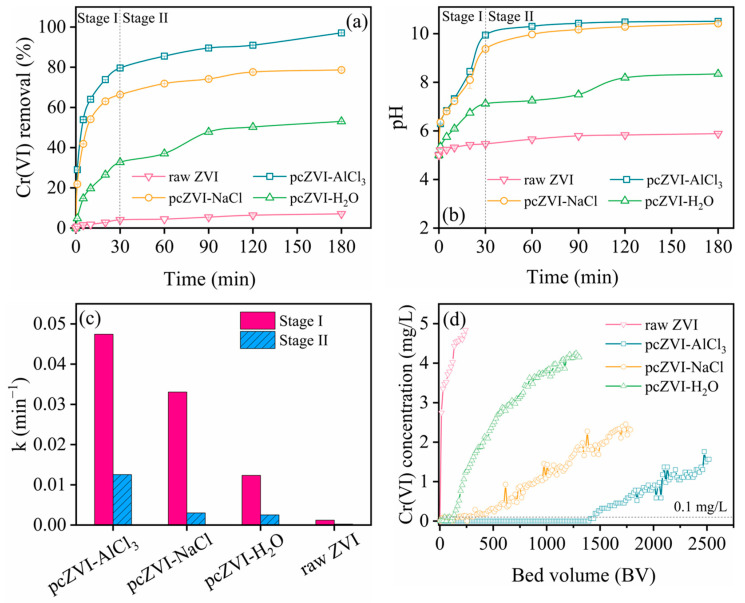
The comparison of Cr(VI) removal by raw ZVI particles and after AlCl_3_ solution, NaCl solution, and DI water pretreatment. (**a**) Cr(VI) removal efficiency variation with time, (**b**) pH variations during the reaction, (**c**) the Cr(VI) removal rate constant k under different conditions, and (**d**) column experiments. Pretreatment conditions: AlCl_3_ = 0.5 mM, NaCl = 1.5 mM, preconditioning time = 4 h, ZVI = 2.5 g/L. Test conditions: Cr(VI) = 10 mg/L, initial pH = 5.0 ± 0.1, T = 25 °C. Column experiment: flow rate = 1 mL/min, EBCT = 4 min.

To elucidate the individual contributions of different components in the AlCl_3_ solution to ZVI pre-corrosion, a comparison was made between the Cr(VI) removal efficiency and reaction rate of various pretreated ZVI samples. Figure 2a reveals that the Cr(VI) removal process in the four systems is categorized into two stages based on pH change and Cr(VI) removal kinetics: an initial rapid Cr(VI) capture within the first 30 min (Stage I), followed by a gradual increase in removal efficiency until equilibrium (Stage II). In addition, the order of Cr(VI) removal capacity is as follows: pcZVI-AlCl_3_ > pcZVI-NaCl > pcZVI-H_2_O > raw ZVI. The significant increase in pcZVI-AlCl_3_ (97.10%) and raw ZVI (7.02%) highlighted the effectiveness of ZVI pre-corrosion in enhancing Cr(VI) removal capacity. In addition, ZVI pretreated with NaCl solution exhibited superior Cr(VI) removal performance and produced more corrosion products than those pretreated with DI water, implying the crucial role of Cl^−^ in accelerating iron corrosion through pitting corrosion. Meanwhile, a lower Cr(VI) removal efficiency of pcZVI-NaCl compared with pcZVI-AlCl_3_ suggests that Al^3+^ generated an acidic condition for ZVI pre-corrosion and also played a significant role in enhancing Cr(VI) removal.

The pH variations during the Cr(VI) removal process by the four kinds of pretreated ZVI were also monitored, as shown in Figure 2b. The solution pH increased sharply in stage I and exhibited a gradual rise in stage II. The pH variation of the four systems also followed the sequence of pcZVI-AlCl_3_ > pcZVI-NaCl > pcZVI-H_2_O > raw ZVI. These results were in accordance with Cr(VI) removal performance in Figure 2a, and the reaction between pretreated ZVI and Cr(VI), O_2_, or protons mainly led to the pH increase [33].

Figure 2c shows the reaction rate (from the first-order kinetic model fitting in Figure S4) comparison of the above preconditioned ZVI. It was clear that the Cr(VI) removal rate in the two stages followed the sequence of pcZVI-AlCl_3_ > pcZVI-NaCl > pcZVI-H_2_O > raw ZVI. Additionally, the Cr(VI) removal rate of pcZVI-AlCl_3_ (0.04744 min^−1^) in stage I was 38.5 times that of raw ZVI (0.0012 min^−1^), indicating corrosion products on the ZVI surface remarkably enhanced the Cr(VI) removal capability.

#### 2.2.2. Column Experiment Comparison and Longevity Evaluation

In this study, the effluent concentration limit of 0.1 mg/L was established with an inflow of 10 mg/L Cr(VI). As illustrated in Figure 2d, the treatment capacity of the four systems followed the order: pcZVI-AlCl_3_ > pcZVI-NaCl > pcZVI-H_2_O > raw ZVI. These results demonstrated that pre-corrosion of ZVI not only enhanced its longevity in continuous-flow processes but also improved the capacity toward Cr(VI) sequestration. Notably, the effluent from the pcZVI-AlCl_3_ system remained below the 0.1 mg/L threshold throughout the 1400 bed volumes. These results suggest that the pcZVI-AlCl_3_ system had significant potential for field applications involving Cr(VI) removal.

#### 2.2.3. SEM-EDS, XRD, and Zeta Potential Analysis

**Figure 3:**
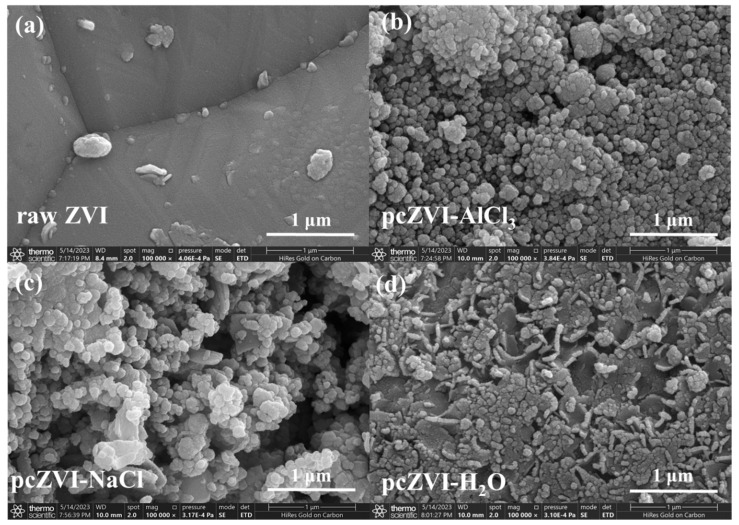
SEM images of raw ZVI particles before (**a**) and after pretreatment by AlCl_3_ solution (**b**), NaCl solution (**c**), and DI water (**d**).

Figure 3 provides a morphological comparison of various pretreated ZVI samples, revealing distinct surface characteristics. The raw ZVI exhibited a relatively smooth surface. In contrast, the surface of pcZVI-AlCl_3_ was covered with spherical particles ranging from 50 to 200 nm in diameter. On the other hand, the pcZVI-NaCl surface displayed both flake-like and spherical particles, indicating a different composition of corrosion products compared to pcZVI-AlCl_3_. The surface of pcZVI-H_2_O appeared partially corroded with thin and irregular pieces. Notably, minimal corrosion products can be peeled off from pcZVI-H_2_O via sonication for collection and, therefore, were not compared with pcZVI-AlCl_3_ and pcZVI-NaCl in the subsequent XRD and Zeta potential analyses. Overall, the flower-like structure in pcZVI-AlCl_3_, pcZVI-NaCl, and pcZVI-H_2_O resulted from ZVI pre-oxidation and contributed to a faster Cr(VI) removal rate compared with raw ZVI.

**Figure 4:**
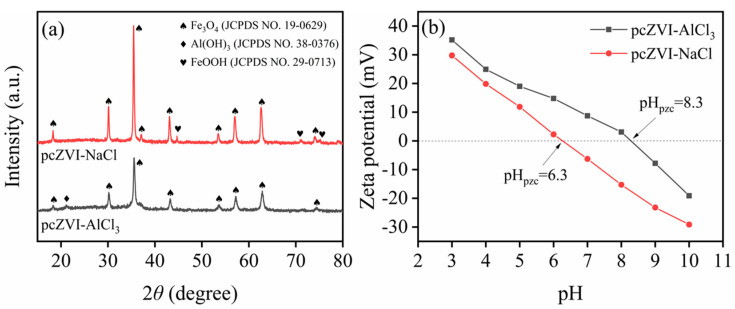
XRD patterns (**a**) and Zeta potential (**b**) of separated corrosion products in pcZVI-AlCl_3_ and pcZVI-NaCl.

Figure 4a shows the XRD pattern comparison between corrosion products in pcZVI-AlCl_3_ and pcZVI-NaCl. Similar to pcZVI-AlCl_3_ (see Section 2.1.2), the typical peaks representing Fe_3_O_4_ also appeared in pcZVI-NaCl, while the diffraction peaks of Al(OH)_3_ were not found. Additionally, the new peaks at 44.8°, 71.2°, and 75.2° were in line with (131), (132), and (331) planes of goethite (JCDPS No. 29-0713). This result was consistent with SEM characterization, indicating the acidic condition in the AlCl_3_ solution significantly influenced the morphology and composition of ZVI corrosion products, ultimately affecting its Cr(VI) removal performance and longevity.

Figure 4b shows the Zeta potential comparison (in a pH range of 3–10) between corrosion products in pcZVI-AlCl_3_ and pcZVI-NaCl. The pH_pzc_ of corrosion products in pcZVI-AlCl_3_ (8.3) was higher than that in pcZVI-NaCl (6.3), which probably resulted from the formation of Al(OH)_3_ [34]. A high value of pH_pzc_ on the surface of pcZVI-AlCl_3_ contributed to capturing negatively charged chromate via electrostatic attraction, which was beneficial for further reaction towards Cr(VI) sequestration.

### 2.3. The Contribution of Corrosion Products in pcZVI-AlCl_3_

#### 2.3.1. The Comparison of Cr(VI) Removal in Different Systems

**Figure 5:**
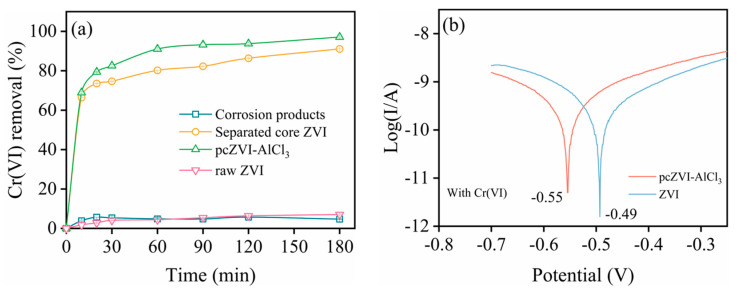
Cr(VI) removal in different systems (**a**) and Tafel analysis comparison of raw ZVI and pcZVI-AlCl_3_ (**b**).

To differentiate the contribution of core ZVI and corrosion products in Cr(VI) removal by pcZVI-AlCl_3_, they were individually separated via sonication for the following comparison, as exhibited in Figure 5a. The Cr(VI) removal efficiency of pcZVI-AlCl_3_ was remarkably higher than the sum of using bare raw ZVI and corrosion products alone, suggesting a synergistic effect between these components.

The removal efficiencies of separated core ZVI and corrosion products after a 180 min reaction were 91.08% and 4.69%, respectively. This result implied that reduction and precipitation were the main pathways of Cr(VI) sequestration by pcZVI-AlCl_3_, and the role of adsorption was less. Compared with raw ZVI, the Cr(VI) removal efficiency of separated core ZVI from pcZVI-AlCl_3_ was significantly improved by 12 times. This was due to the passivation layer on raw ZVI being destroyed via pre-corrosion. Figure S5 shows the surface of the separated core ZVI was still partially covered by some corrosion products, which probably promoted electron transfer for Cr(VI) removal.

#### 2.3.2. Tafel Analysis

Figure 5b shows the open-circuit potential comparison via Tafel scans between raw ZVI and pcZVI-AlCl_3_ in the Cr(VI) solution. The potential of pcZVI-AlCl_3_ (−0.55 V) was lower than ZVI (−0.49 V), suggesting a lower corrosion resistance of pcZVI-AlCl_3_. This resulted from the semi-conductive Fe_3_O_4_ on the ZVI surface, which facilitated sustainable electron transfer for Cr(VI) reduction.

### 2.4. Effect of Solution Chemistry on Cr(VI) Removal

#### 2.4.1. Effect of Initial pH

**Figure 6:**
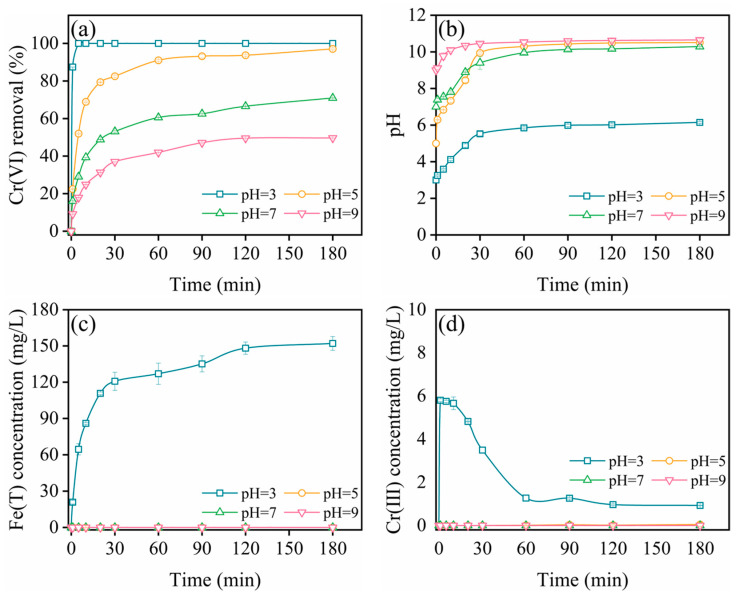
The effect of initial pH on Cr(VI) removal by pcZVI-AlCl_3_ (**a**), and the corresponding pH (**b**) and total iron (**c**) and Cr(III) (**d**) concentration variations during the reaction process. Pretreatment conditions: AlCl_3_ = 0.5 mM, preconditioning time = 4 h, ZVI = 2.5 g/L. Test conditions: initial pH = 3–9, Cr(VI) = 10 mg/L, T = 25 °C.

Figure 6a illustrates the efficacy of pcZVI-AlCl_3_ in removing Cr(VI) across a pH spectrum of 3–9. Notably, at pH 3, the removal efficiency for Cr(VI) approached 100%, surpassing that of other pH conditions, and gradually declined with increasing pH. The solution pH experienced an initial rise within the first 30 min and stabilized thereafter, likely attributed to ZVI corrosion, as depicted in Figure 6b. This process yielded Fe(II) and Fe(III) in the form of iron oxides, which passivated the ZVI. These findings align with prior research and conform to the two-stage reaction process detailed in Section 2.2.1 [33,35].

In Figure 6c, variations in Fe(T) concentration are depicted. At pH 3, ZVI corrosion ensued vigorously, generating substantial Fe(II) due to the presence of high levels of H^+^, while total iron concentration approached zero at pH 5–9, indicative of iron hydroxide formation. The release of Fe(II) facilitated Cr(VI) reduction, resulting in a sharp rise followed by a decline in Cr(III) concentration under pH 3, as demonstrated in Figure 6d. Consequently, the precipitation of Fe(III)-Cr(III) or Cr(III) hydroxides on the pcZVI-AlCl_3_ surface hindered further reaction between ZVI and Cr(VI). The corresponding reactions are outlined below:

(1) $${\mathrm{Fe}}^{0}+{{\mathrm{CrO}}_{4}}^{2-}+H^{+}\to {\mathrm{Fe}}^{3+}+{\mathrm{Cr}}^{3+}+4H_{2}O$$

(2) $$3{\mathrm{Fe}}^{2+}+{\mathrm{CrO}}_{4}^{2-}+8H^{+}\to 3{\mathrm{Fe}}^{3+}+{\mathrm{Cr}}^{3+}+4H_{2}O$$

(3) $${\mathrm{xCr}}^{3+}+(1-x){\mathrm{Fe}}^{3+}+3H_{2}O\to {\mathrm{Cr}}_{x}{\mathrm{Fe}}_{1-x}{\left(\mathrm{OH}\right)}_{3}+3H^{+}$$

#### 2.4.2. Matrix Effects

The impact of Cl^−^, NO_3_^−^, SO_4_^2−^, HCO_3_^−^, Ca^2+^, and Mg^2+^ on Cr(VI) removal by pcZVI-AlCl_3_ was explored, as demonstrated in Figure S6. The presence of Ca^2+^ and Mg^2+^ slightly improved the Cr(VI) removal efficiency, presumably due to coprecipitation in an alkaline environment. This finding indicated that pcZVI-AlCl_3_ can effectively remove Cr(VI), even in conditions of high water hardness. On the other hand, the Cr(VI) removal was not significantly disturbed by Cl^−^. However, NO_3_^−^, SO_4_^2−^, and HCO_3_^−^ exhibited suppressive effects. Although NO_3_^−^ competed with Cr(VI) for electrons, it had the least inhibitory effect. SO_4_^2−^ affected Cr(VI) removal due to its anionic structure similar to that of adsorbed species. HCO_3_^−^ showed a severe inhibitory effect, presumably resulting from FeCO_3_ precipitation, similar to the behavior observed for ZVI [36]. The inhibitory effect of HCO_3_^−^ and SO_4_^2−^ could also be ascribed to the complexation of the corrosion products of pcZVI-AlCl_3_, leading to a less positively charged surface [37].

### 2.5. Mechanisms

#### 2.5.1. SEM-EDS and XRD

**Figure 7:**
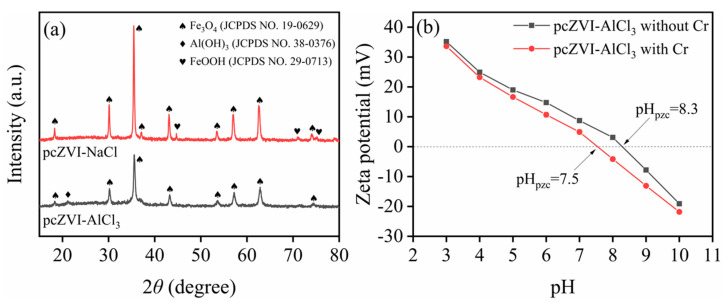
XRD patterns (**a**) and Zeta potential (**b**) of separated corrosion products in pcZVI-AlCl_3_ before and after Cr(VI) removal.

Figure S7 shows the SEM images and EDS of pcZVI-AlCl_3_ after Cr(VI) removal. The morphology was close to that prior to the reaction, and the Cr element appeared, indicating the immobilization of Cr by pcZVI-AlCl_3_. Figure 7a exhibits the XRD patterns of corrosion products in pcZVI-AlCl_3_, compared before and after the reaction. A new peak at 33.3° was observed in the corrosion products following Cr(VI) removal, which was probably ascribed to the (130) plane of goethite (JCPDS No. 29-0713). This finding indicates that some Fe_3_O_4_ had been transformed into FeOOH through oxidation. However, no peaks corresponding to Cr(III) precipitates were detected in the corrosion products after the reaction, which could be due to the amorphous structure of these precipitates.

#### 2.5.2. Zeta Potential

Figure 7b shows the Zeta potential of corrosion products before and after Cr(VI) removal by pcZVI-AlCl_3_. The pH_pzc_ of corrosion products decreased from 8.3 to 7.5 after Cr(VI) removal. This decrease in pH_pzc_ was likely attributed to the adsorption of Cr(VI) on the corrosion products via ligand exchange [34,38], which supported that adsorption contributed to negatively charged Cr(VI) species removal by pcZVI-AlCl_3_.

#### 2.5.3. XPS

**Figure 8:**
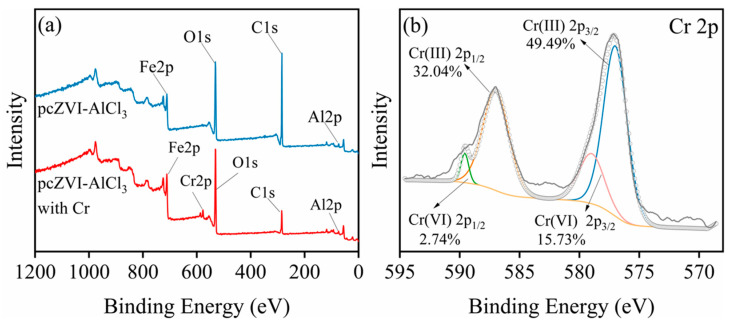
The wide-scan XPS spectra of separated corrosion products in pcZVI-AlCl_3_ before and after Cr(VI) removal (**a**), and Cr 2p XPS spectra of corrosion products after the reaction (**b**).

Figure 8a displays the wide-scan XPS spectra within the energy range of 0 to 1200 eV. The peaks observed at 73.65 eV, 283.81 eV, 531.12 eV, and 711.11 eV correspond to Al 2p, C 1s, O 1s, and Fe 2p, respectively. Notably, after Cr(VI) removal, a new peak emerged at 577.14 eV, which is in line with Cr. These results confirmed the successful immobilization of Cr on corrosion products and were consistent with the findings obtained from the EDS characterization.

Figure 8b shows the Cr 2p XPS spectra of corrosion products after the reaction. The peaks at 589.58 eV and 579.00 eV correspond to Cr(VI) 2p_1/2_ and Cr(VI) 2p_3/2_, respectively. These peaks confirmed the presence of adsorbed negative Cr(VI) species on the surface of the corrosion products. [33]. Additionally, the peaks at 586.98 eV and 577.04 eV were attributed to Cr(III) 2p_1/2_ and Cr(III) 2p_3/2_, respectively. This result indicated that Cr(VI) was reduced to Cr(III) during the process [35]. In addition, Table S2 reveals that the proportion of Cr(III) is 81.53%, while Cr(VI) accounts for 18.47%. This result indicates that both adsorption and reduction contributed significantly to the overall Cr(VI) removal process [39]. Furthermore, the precipitation of Cr(III) species participated in the removal of Cr(VI).

**Figure 9:**
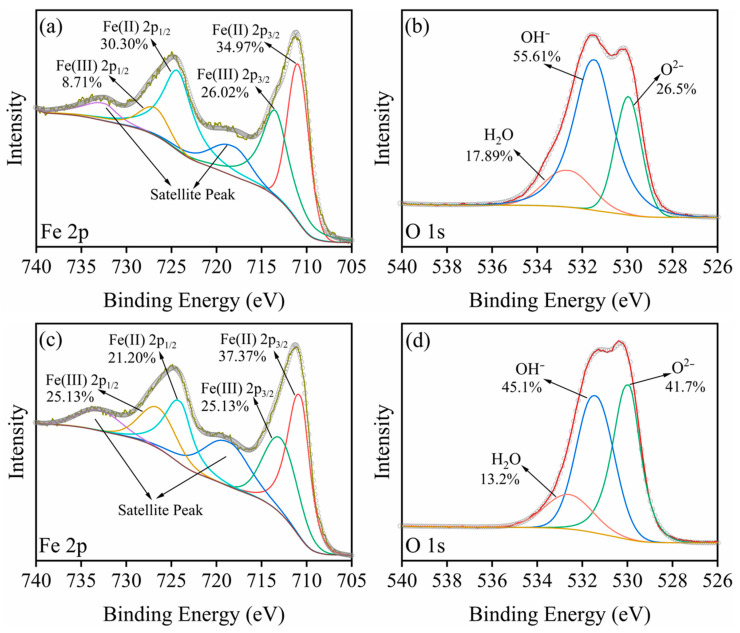
The Fe 2p and O 1s high-resolution XPS spectra of separated corrosion products in pcZVI-AlCl_3_ before (**a**,**b**) and after (**c**,**d**) Cr(VI) removal.

Figure 9a,c show the Fe 2p XPS spectra of corrosion products before and after Cr(VI) removal. These spectra reveal that both Fe(II) and Fe(III) species existed in the corrosion products. A detailed analysis of the binding energies and proportions of the corresponding Fe(II) 2p_1/2_, Fe(II) 2p_3/2_, Fe(III) 2p_1/2_, and Fe(III) 2p_3/2_ peaks is provided in Table S3. Notably, the content of Fe(II) in the corrosion products declined from 65.27% to 58.57% after Cr(VI) removal. This decrease suggests that partial Fe(II) species were oxidized to Fe(III) during the Cr(VI) removal process [40].

Figure 9b,d compare the O 1s spectra of corrosion products before and after Cr(VI) removal. The peaks at 531.40 eV, 530.00 eV, and 532. 60 eV fitted well with OH^−^, O^2−^, and H_2_O species, respectively. As depicted in Table S4, the proportion of OH^−^ decreased and O^2−^ increased after Cr(VI) removal. This trend resulted from the adsorption of negatively charged Cr(VI) species on the corrosion products, as well as the precipitation of Cr(III) species. The adsorption of Cr(VI) likely displaced some OH^−^ groups (ligand exchange), leading to a decrease in their relative proportion. Concurrently, the Cr(III) precipitates may result in the Cr(III)-related hydroxides, thereby increasing the proportion of O^2−^ species [41]. These findings provided further evidence for Cr(VI) removal by pcZVI-AlCl_3_ and the associated changes in the surface chemistry of the corrosion products.

Overall, the Cr(VI) removal by the pcZVI-AlCl_3_ process included several steps. The adsorption occurred in the initial stage via electrostatic attraction and ligand exchange, and this process was enhanced by the Al(OH)_3_ in the corrosion products with a high pH_pzc_ of 8.3. The semi-conductive Fe_3_O_4_ on the ZVI surface constructed a core-shell-like galvanic cell to enhance electron transfer from ZVI to the surface of pcZVI-AlCl_3_ for Cr(VI) reduction. The aqueous Fe(II), surface-bound Fe(II), and structural Fe(II) could reduce Cr(VI) to Cr(III), and these processes are highly dependent on pH. The released Fe(II) was oxidized to Fe(III) and participated in the coprecipitation with Cr(III).

## 3. Materials and Methods

### 3.1. Chemicals and Materials

The detailed chemicals used in this study are shown in Text S1 of Supplementary Data.

### 3.2. Preparation and Optimization of pcZVI-AlCl3

To achieve the pre-corrosion of micron-scale ZVI, 50 mL centrifuge tubes were prepared by filling them with raw ZVI powder and 40 mL of AlCl_3_ solution. These tubes were then rotated at a speed of 30 rpm for a specified duration to allow the reaction to proceed. Once the pretreatment was complete, the suspension and pretreated ZVI were separated using a magnet. The mixed material was subsequently washed with DI water until the conductivity of the effluent stabilized, followed by placement in a vacuum environment.

To obtain the pretreated ZVI for maximum Cr(VI) removal, various parameters influencing the pre-corrosion process were examined. Firstly, dosages of ZVI ranging from 0.5 to 2.5 g/L were tested to determine the optimal amount. Secondly, pre-corrosion time was varied from 1 to 12 h to establish the most favorable corrosion extent. Lastly, the impact of initial Al^3+^ concentrations (0.1 to 1 mM) and dissolved oxygen (DO) levels (0.5 to 10 mg/L) were assessed. To control initial DO concentrations, the solution was bubbled with nitrogen for several minutes, and the experiment was conducted in an anaerobic chamber.

### 3.3. Batch Experiments

In the subsequent experiments, the optimized pcZVI-AlCl_3_ was utilized. All the experiments were conducted at least in duplicate, and an average was used. Unless otherwise stated, the experimental solutions contained 10 mg/L Cr(VI) (pH = 5 ± 0.1). Forty milliliters of the Cr solution was dispensed into a series of centrifuge tubes containing pre-corroded ZVI. These tubes were then rotated at 30 rpm at 25 °C. At predefined time intervals, a set of tubes was removed for immediate pH measurement. The filtrate was tested for Cr(VI), Cr(III), and total iron (Fe(T)) concentrations after filtration with 0.45 µm PVDF membranes and syringes. To assess the initial pH effects, experiments were conducted with solutions ranging from pH 3 to 9. Additionally, matrix effects were examined by introducing Cl^−^, SO_4_^2−^, NO_3_^−^, HCO_3_^−^, Ca^2+^, and Mg^2+^ in concentrations ranging from 0 to 10 mM.

To elucidate the specific contribution of different components in the AlCl_3_ solution to ZVI corrosion, ZVI pretreated with DI water and with NaCl solution were established as comparative controls for Cr(VI) removal. These pretreated ZVIs were designated as pcZVI-AlCl_3_, pcZVI-NaCl, and pcZVI-H_2_O, respectively. Additionally, raw ZVI was employed for Cr(VI) removal under identical conditions to serve as a control test. To further investigate the individual influence of constituents in pcZVI-AlCl_3_ on Cr(VI) removal, the corrosion products and ZVI core were separated via sonication and tested separately for their Cr(VI) removal capabilities.

### 3.4. Characterization of Preconditioned ZVI and Corrosion Products

The optimized pre-corroded ZVI was evaporated at 35 °C in a vacuum oven (DZF-6030A, Yiheng, China) to ensure stability and consistency before being characterized. To separate the corrosion products from the surface of pre-corroded ZVI, sonication (SB-5200DT, SCIENTZ, Ningbo, China) was performed for 15 min. This was followed by centrifugation (TG16-WS, Xiangyi, Xiangtan, China) at 8000 rpm for 10 min to effectively separate the corrosion products. Both the pre-corroded ZVI and the separated corrosion products were then dried under the same vacuum conditions. All samples were stored in self-sealing plastic bags to ensure purity and prevent oxidation before characterization. The detailed characterization of pretreated ZVI and corrosion products are presented in Text S2 of Supplementary Data, including SEM-EDS, XRD, Raman, and ^57^Fe Mössbauer spectra, Zeta potential, and XPS. It should be noted that the pre-corroded ZVI particles were used for SEM and ^57^Fe Mössbauer spectra detection, while the separated corrosion products via sonication were used for XRD, Raman, Zeta potential, and XPS analysis.

### 3.5. Continuous Flow Experiments

To compare the durability of ZVI following various pretreatment methods (AlCl_3_ solution, NaCl solution, and DI water), the continuous-flow setup was conducted. The raw ZVI was used as a control for comparison. These experiments aimed to simulate real-world conditions and assess their longevity in a controlled environment. Figure S1 provides a digital photograph of these column experiments. The inner diameter and height of the column are 16 mm and 300 mm, respectively. In these experiments, 10 g of raw ZVI powder was pretreated under optimized conditions and then loaded into the column, filling approximately 2 cm of the column’s height. The use of quartz sand and fiber filter served to support the pretreated ZVI and prevent the leakage of corrosion products. The Cr(VI) solution entered into the system through a peristaltic pump (1 mL/min) in an up-flow mode. An automatic sampler was used for effluent collection, followed by subsequent Cr(VI) concentration analysis.

### 3.6. Analytical Methods

The detailed analytical methods used in this study are presented in Text S3 of Supplementary Data.

## 4. Conclusions

In this study, the ZVI pre-corrosion with AlCl_3_ solution for enhanced Cr(VI) removal and longevity was systematically explored. The optimal conditions for the preconditioning process were determined to be 2.5 g/L ZVI, 0.5 mM AlCl_3_, and a preconditioning time of 4 h. The components in the corrosion products of optimized pcZVI-AlCl_3_ mainly included Fe_3_O_4_, FeOOH, FeO, and Al(OH)_3_, with Fe_3_O_4_ being the dominant component. The formation of Al(OH)_3_ raised the pH_pzc_ of the corrosion products from 6.3 to 8.3, facilitating the effective capture of negatively charged Cr(VI) species. Among the various pretreatment methods tested, the order of Cr(VI) removal capacity and longevity of ZVI were as follows: pcZVI-AlCl_3_ > pcZVI-NaCl > pcZVI-H_2_O > raw ZVI. The pcZVI-AlCl_3_ system exhibited potential for Cr(VI) removal in field applications, which maintained the effluent concentration below 0.1 mg/L for more than 1400 BV. The acidic condition was beneficial to Cr(VI) removal. The results of matrix effects showed that pcZVI-AlCl_3_ removed Cr(VI) effectively in a high-hardness environment, while NO_3_^−^, SO_4_^2−^, and HCO_3_^−^ showed suppressive effects on Cr(VI) removal. A synergistic effect existed between corrosion products and ZVI for enhanced electron transfer from core ZVI towards Cr(VI). The removal mechanisms included adsorption (mainly via electrostatic attraction and ligand exchange), reduction of Cr(VI) to Cr(III), and precipitation of Fe(III)-Cr(III) or Cr(III) hydroxide. Adsorption played a lesser role than reduction and precipitation.

## Figures and Tables

**Table 1:** The meanings of the symbols.

Symbols	Meanings
ZVI (Fe^0^)	Zero-valent iron
pcZVI-AlCl_3_	ZVI pretreated with AlCl_3_ solution
pcZVI-NaCl	ZVI pretreated with NaCl solution
pcZVI-H_2_O	ZVI pretreated with DI water
Cr(IV)	Hexavalent chromium
Cr(III)	Trivalent chromium
Fe(T)	Total iron
DO	Dissolved oxygen
pH_pzc_	pH point of zero charge
SEM-EDS	Scanning electron microscopy with X-ray energy dispersive spectrometer
XRD	X-ray diffraction spectrometer
XPS	X-ray photoelectron spectroscopy

## Data Availability

The original contributions presented in the study are included in the article/Supplementary Materials, further inquiries can be directed to the corresponding author/s.

## Supplementary Materials

The following supporting information can be downloaded at: https://www.mdpi.com/article/10.3390/molecules29102350/s1, Texts S1–S4, Figure S1: The digital photo of the column experiments in a continuous-flow mode; Figure S2: Effect of ZVI dosage (a), pre-corrosion time (b), initial AlCl_3_ concentration (c), and DO concentration (d) on ZVI pretreatment for Cr(VI) removal; Figure S3: The effect of initial Cr(VI) concentration on Cr(VI) removal efficiency by raw ZVI and after AlCl_3_ solution, NaCl solution, and DI water pretreatment.; Figure S4: The first-order kinetic model fitting of Cr(VI) removal by raw ZVI and after AlCl_3_ solution, NaCl solution, and DI water pretreatment; Figure S5: SEM images of the pcZVI-AlCl_3_ surface with corrosion products peeled off via sonication; Figure S6: Effect of co-existing cations and anions on Cr(VI) removal by pcZVI-AlCl_3_; Figure S7: SEM images (a–c), EDS (d), and Fe, O, Al, and Cr elemental mappings (e) of pcZVI-AlCl_3_ after Cr(VI) removal; Table S1: The fitting parameters of ^57^Fe Mössbauer spectra; Table S2: Relative contents of Cr 2p in different chemical states of corrosion products in pcZVI after Cr(VI) removal; Table S3: Relative contents of Fe 2p in different chemical states of corrosion products in pcZVI before and after Cr(VI) removal; Table S4: Relative contents of O 1s in different chemical states of corrosion products in pcZVI before and after Cr(VI) removal. References [42,43] are cited in the Supplementary Materials.

## References

[1] Fang Y., Wu X., Dai M., Lopez-Valdivieso A., Raza S., Ali I., Peng C., Li J., Naz I. (2021). The sequestration of aqueous Cr(VI) by zero valent iron-based materials: From synthesis to practical application. J. Clean. Prod..

[2] Su M., Fang Y., Li B., Yin W., Gu J., Liang H., Li P., Wu J. (2019). Enhanced hexavalent chromium removal by activated carbon modified with micro-sized goethite using a facile impregnation method. Sci. Total Environ..

[3] Zhang L., Fu F., Tang B. (2019). Adsorption and redox conversion behaviors of Cr(VI) on goethite/carbon microspheres and akaganeite/carbon microspheres composites. Chem. Eng. J..

[4] Zhang S., Wu M., Tang T., Xing Q., Peng C., Li F., Liu H., Luo X., Zou J., Min X. (2018). Mechanism investigation of anoxic Cr(VI) removal by nano zero-valent iron based on XPS analysis in time scale. Chem. Eng. J..

[5] (2022). Standards for Drinking Water Quality.

[6] Mallik A.K., Moktadir M.A., Rahman M.A., Shahruzzaman M., Rahman M.M. (2022). Progress in surface-modified silicas for Cr (VI) adsorption: A review. J. Hazard. Mater..

[7] Yu W., Gan Z., Wang J., Zhao Y., Han J., Fang L., Wei X., Qiu Z., Zhu B. (2021). A novel negatively charged nanofiltration membrane with improved and stable rejection of Cr (VI) and phosphate under different pH conditions. J. Membr. Sci..

[8] Wang C., Ren X., Wang P., Chang C. (2022). The state of the art review on photocatalytic Cr (VI) reduction over MOFs-based photocatalysts: From batch experiment to continuous operation. Chemosphere.

[9] Peng H., Guo J. (2020). Removal of chromium from wastewater by membrane filtration, chemical precipitation, ion exchange, adsorption electrocoagulation, electrochemical reduction, electrodialysis, electrodeionization, photocatalysis and nanotechnology: A review. Environ. Chem. Lett..

[10] Stern C.M., Jegede T.O., Hulse V.A., Elgrishi N. (2021). Electrochemical reduction of Cr (VI) in water: Lessons learned from fundamental studies and applications. Chem. Soc. Rev..

[11] Lu J., Zhang B., He C., Borthwick A.G. (2020). The role of natural Fe (II)-bearing minerals in chemoautotrophic chromium (VI) bio-reduction in groundwater. J. Hazard. Mater..

[12] Li H., Qian L., Liang C., Zheng T., Dong X., Chen M. (2023). Enhanced Cr(VI) reduction by zero-valent iron and ferroferric oxide wet ball milling: Synergy of electron storage and electron transfer. Chem. Eng. J..

[13] Yin W., Li Y., Wu J., Chen G., Jiang G., Li P., Gu J., Liang H., Liu C. (2017). Enhanced Cr(VI) removal from groundwater by Fe^0^-H_2_O system with bio-amended iron corrosion. J. Hazard. Mater..

[14] Ai H., Zhang K., Penn C.J., Zhang H. (2023). Phosphate removal by low-cost industrial byproduct iron shavings: Efficacy and longevity. Water Res..

[15] Dai Y., Duan L., Dong Y., Zhao W., Zhao S. (2022). Elemental sulfur generated in situ from Fe(III) and sulfide promotes sulfidation of microscale zero-valent iron for superior Cr(VI) removal. J. Hazard. Mater..

[16] Gan R., Ye Y., Zhan Z., Zhang Q., Deng Y., Liu Y., Li H., Wan J., Pei X., Li Q. (2024). One-step strategy for efficient Cr(VI) removal via phytate modified zero- valent iron: Accelerated electron transfer and enhanced coordination effect. J. Hazard. Mater..

[17] Wang W., Hu B., Wang C., Liang Z., Cui F., Zhao Z., Yang C. (2019). Cr(VI) removal by micron-scale iron-carbon composite induced by ball milling: The role of activated carbon. Chem. Eng. J..

[18] Ou J., Sheu Y., Tsang D.C.W., Sun Y., Kao C. (2020). Application of iron/aluminum bimetallic nanoparticle system for chromium-contaminated groundwater remediation. Chemosphere.

[19] Mu Y., Ai Z., Zhang L., Song F. (2015). Insight into core-shell dependent anoxic Cr(VI) removal with Fe@Fe_2_O_3_ nanowires: Indispensable role of surface bound Fe(II). ACS Appl. Mater. Interfaces.

[20] Hu Y., Zhang M., Li X. (2019). Improved longevity of nanoscale zero-valent iron with a magnesium hydroxide coating shell for the removal of Cr(VI) in sand columns. Environ. Int..

[21] Luo X., Guo X., Xia X., Zhang X., Ma N., Leng S., Ullah S., Ayalew Z.M. (2020). Rapid and long-effective removal of phosphate from water by zero-valent iron in combination with hypochlorite (ZVI/NaClO). Chem. Eng. J..

[22] Shan C., Chen J., Yang Z., Jia H., Guan X., Zhang W., Pan B. (2018). Enhanced removal of Se(VI) from water via pre-corrosion of zero-valent iron using H_2_O_2_/HCl: Effect of solution chemistry and mechanism investigation. Water Res..

[23] Xu L., Huang Y. (2019). A simple and novel method to enhance As (V) removal by zero valent iron and activated iron media through air injection at intervals. Chemosphere.

[24] Sleiman N., Deluchat V., Wazne M., Mallet M., Courtin-Nomade A., Kazpard V., Baudu M. (2017). Phosphate removal from aqueous solutions using zero valent iron (ZVI): Influence of solution composition and ZVI aging. Colloids Surf. A Physicochem. Eng. Asp..

[25] Yang Z., Xu H., Shan C., Jiang Z., Pan B. (2017). Effects of brining on the corrosion of ZVI and its subsequent As(III/V) and Se(IV/VI) removal from water. Chemosphere.

[26] Tang C., Wang X., Zhang Y., Liu N., Hu X. (2024). Corrosion behaviors and kinetics of nanoscale zero-valent iron in water: A review. J. Environ. Sci..

[27] Xu R., Zhang M., Mortimer R.J.G., Pan G. (2017). Enhanced Phosphorus Locking by Novel Lanthanum/Aluminum-Hydroxide Composite: Implications for Eutrophication Control. Environ. Sci. Technol..

[28] Yang Y., Gai W., Zhou J., Deng Z. (2020). Surface modified zero-valent aluminum for Cr(VI) removal at neutral pH. Chem. Eng. J..

[29] Liang L., Guan X., Shi Z., Li J., Wu Y., Tratnyek P.G. (2014). Coupled effects of aging and weak magnetic fields on sequestration of selenite by zero-valent iron. Environ. Sci. Technol..

[30] Nieuwoudt M.K., Comins J.D., Cukrowski I. (2011). The growth of the passive film on iron in 0.05 M NaOH studied in situ by Raman micro-spectroscopy and electrochemical polarisation. Part I: Near-resonance enhancement of the Raman spectra of iron oxide and oxyhydroxide compounds. J. Raman Spectrosc..

[31] Huang E., Li A., Xu J.A., Chen R.J., Yamanaka T. (1996). High-pressure phase transition in Al(OH)_3_: Raman and X-ray observations. Geophys. Res. Lett..

[32] Liu A., Liu J., Zhang W. (2015). Transformation and composition evolution of nanoscale zero valent iron (nZVI) synthesized by borohydride reduction in static water. Chemosphere.

[33] Zhang L., Fu F., Ding Z., Pang J. (2017). Rapid removal of aqueous Cr(VI) and the removal mechanism using ZVI/Fe_3_O_4_/Fe^2+^ system. Desalin. Water Treat..

[34] Shi W., Fu Y., Jiang W., Ye Y., Kang J., Liu D., Ren Y., Li D., Luo C., Xu Z. (2019). Enhanced phosphate removal by zeolite loaded with Mg–Al–La ternary (hydr)oxides from aqueous solutions: Performance and mechanism. Chem. Eng. J..

[35] Du J., Bao J., Lu C., Werner D. (2016). Reductive sequestration of chromate by hierarchical FeS@Fe^0^ particles. Water Res..

[36] Lo I.M., Lam C.S., Lai K.C. (2006). Hardness and carbonate effects on the reactivity of zero-valent iron for Cr (VI) removal. Water Res..

[37] Chen G., Hofstetter T.B., Gorski C.A. (2020). Role of Carbonate in Thermodynamic Relationships Describing Pollutant Reduction Kinetics by Iron Oxide-Bound Fe^2+^. Environ. Sci. Technol..

[38] Wu B., Fang L., Fortner J.D., Guan X., Lo I.M. (2017). Highly efficient and selective phosphate removal from wastewater by magnetically recoverable La(OH)_3_/Fe_3_O_4_ nanocomposites. Water Res..

[39] He Y., Lin H., Luo M., Liu J., Dong Y., Li B. (2020). Highly efficient remediation of groundwater co-contaminated with Cr(VI) and nitrate by using nano-Fe/Pd bimetal-loaded zeolite: Process product and interaction mechanism. Environ. Pollut..

[40] Cao R., Liu S., Yang X., Wang C., Wang Y., Wang W., Pi Y. (2022). Enhanced remediation of Cr (VI)-contaminated groundwater by coupling electrokinetics with ZVI/Fe_3_O_4_/AC-based permeable reactive barrier. J. Environ. Sci..

[41] Zhu L., Fu F., Tang B. (2018). Coexistence or aggression? Insight into the influence of phosphate on Cr(VI) adsorption onto aluminum-substituted ferrihydrite. Chemosphere.

[42] Rancourt D.G., Ping J.Y. (1991). Voigt-based methods for arbitrary-shape static hyperfine parameter distributions in Mössbauer spectroscopy. Nucl. Instrum. Methods Phys. Res. Sect. B Beam Interact. Mater. At..

[43] Wan J., Wu B., Lo I.M.C. (2020). Development of Fe^0^/Fe_3_O_4_ composites with tunable properties facilitated by Fe^2+^ for phosphate removal from river water. Chem. Eng. J..

