# Supplementary material for: Enhanced Chromium (VI) Removal by Micron-Scale Zero-Valent Iron Pretreated with Aluminum Chloride under Aerobic Conditions
Source: Molecules. 2024 May 16;29(10):2350. doi: 10.3390/molecules29102350 (PMC11123959; doi:10.3390/molecules29102350)
Supplement: Supplementary file 1 [file molecules-29-02350-s001.zip › molecules-3002340-supplementary.pdf]

Supplementary materials for

Enhanced chromium (VI) removal by micron-scale zero-valent iron pretreated with aluminum chloride under aerobic condition

Xuejun Long [1, 2, 3], Rui Li [1], Jun Wan [1, 2, 3, *], Zhenxing Zhong [1, 2, 3, *], Yuxuan Ye [1], Jiazhi Yang [1], Jun Luo [1], Jin Xia [1], Yaomeng Liu [1]

[1] *School of Environmental Engineering, Wuhan Textile University, Wuhan 430200, China*

[2] *State Key Laboratory of New Textile Materials and Advanced Processing Technologies, Wuhan Textile University, Wuhan 430200, China*

[3] *Engineering Research Center of Ministry of Education for Clean Production of Textile Dyeing and Printing, Wuhan Textile University, Wuhan 430200, China*

**Correspondence authors*

E-mail addresses: wanjun@wtu.edu.cn *(J. Wan)*, xing2006z@163.com *(Z. Zhong).*

Number of pages: 18

Number of texts: 4

Number of figures: 7

Number of tables: 4

Text S1

Zero-valent iron powder with a mesh size of 100 was obtained from Aladdin, Shanghai, China. Potassium dichromate ($K_2Cr_2O_7$) of analytical grade, serving as the Cr(VI) source in this study, was purchased from the Tailande Chemical Reagent Factory in Tianjin. All other chemical reagents, also of analytical grade, were sourced from China Sinopharm Chemical Reagent Co., Ltd (Shanghai, China). Deionized water (DI) with a conductivity of 18.25 MΩ·cm was provided by an integrated laboratory water purification system from Aike in Chengdu, China.

Text S2

The morphological characteristics and elemental composition of pcZVI were examined using scanning electron microscopy (SEM, Thermo Scientific, Quattro S, Waltham, MA, USA) combined with an x-ray energy dispersive spectrometer (EDS). An x-ray diffraction spectrometer (XRD, Rigaku, Smartlab 9KW, Tokyo, Japan) was employed to determine the crystal structure of the corrosion products, scanning a 2θ range of 15 to 80 degrees. To further investigate the iron species and related proportion of the pcZVI, ^{57}Fe Mössbauer spectra were conducted by applying a conventional spectrometer (Wissel MS-500, Emmerich, Germany) in the transmission geometry of constant acceleration mode at room temperature. The ^{57}Co(Rh) source with an activity of 25 mCi was used and velocity calibration was performed with an α-Fe absorber. The spectra were fitted by the software Recoil using Voigt-based fitting (VBF) analysis [1]. Raman spectroscopy (Horiba LabRAM HR Evolution, Tokyo, Japan) was used to investigate the structure of iron and aluminum species within the corrosion products, utilizing a 514 nm laser within the range of 50–1000 cm^{-1}. To assess the electrochemical properties of ZVI and pcZVI, Tafel scans were conducted using an electrochemical workstation (CHI 660E, Wuhan Corrtest Instrument, Wuhan, China) within a three-electrode system, following the methods outlined in previous research [2]. To determine the pH_{pzc} (point of zero charge) of the corrosion products, the zeta potential values were measured under various pH conditions using a zeta potential analyzer (Zetasizer Nano-ZS90, Malvern Instruments, Malvern, UK). The 1 mM NaCl solution with pH ranging from 3-10 was used in zeta potential tests. Additionally, X-ray photoelectron spectroscopy (XPS, Escalab 250Xi, Thermo Scientific, Waltham, MA, USA) was employed to analyze the binding energy and chemical states of elements on the

surface of the corrosion products, both before and after Cr(VI) removal. The Avantage software was used to differentiate the peaks in the XPS spectra, with C 1s peak at 284.8 eV serving as a reference for binding energy calibration.

Text S3

Cr(VI) concentration was detected by 1,5-Diphenylcarbohydrazide spectrophotometric method at a wavelength of 540nm with the UV/Vis spectrophotometer (UV-1600PC, Mapada, China). The concentrations of total Cr (Cr(T)), Al^{3+} and total iron (Fe(T)) were also measured by using the inductively coupled plasma–optical emission spectrometer (ICP-OES, ICP2060T, Skyray Instrument, USA). The Cr(III) concentration was calculated according to the difference between Cr(T) and Cr(VI). The pH was detected by a pH meter (FE20K PLUS, Mettler-Toledo, Switzerland), and DO was detected by a portable DO meter (HACH HQ30d, USA).

Text S4

The first-order kinetic model (Eq. (S1)) was applied to fit the Cr(VI) removal kinetics data in raw ZVI, pcZVI-$AlCl_3$, pcZVI-NaCl and pcZVI-H_2O systems.

$$C = C_0 e^{(-kt)} \tag{S1}$$

where C and C_0 are the Cr(VI) concentrations (mg/L) in the beginning and reaction at time t (min), respectively; k is the rate constant for the first-order kinetic model (min^{-1}).

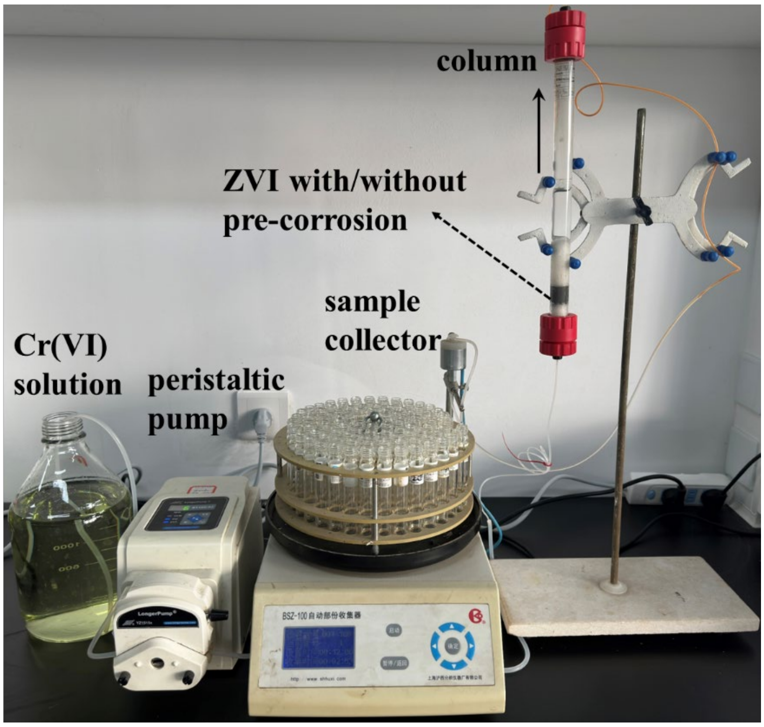

Figure S1. The digital photo of the column experiments in a continuous-flow mode.

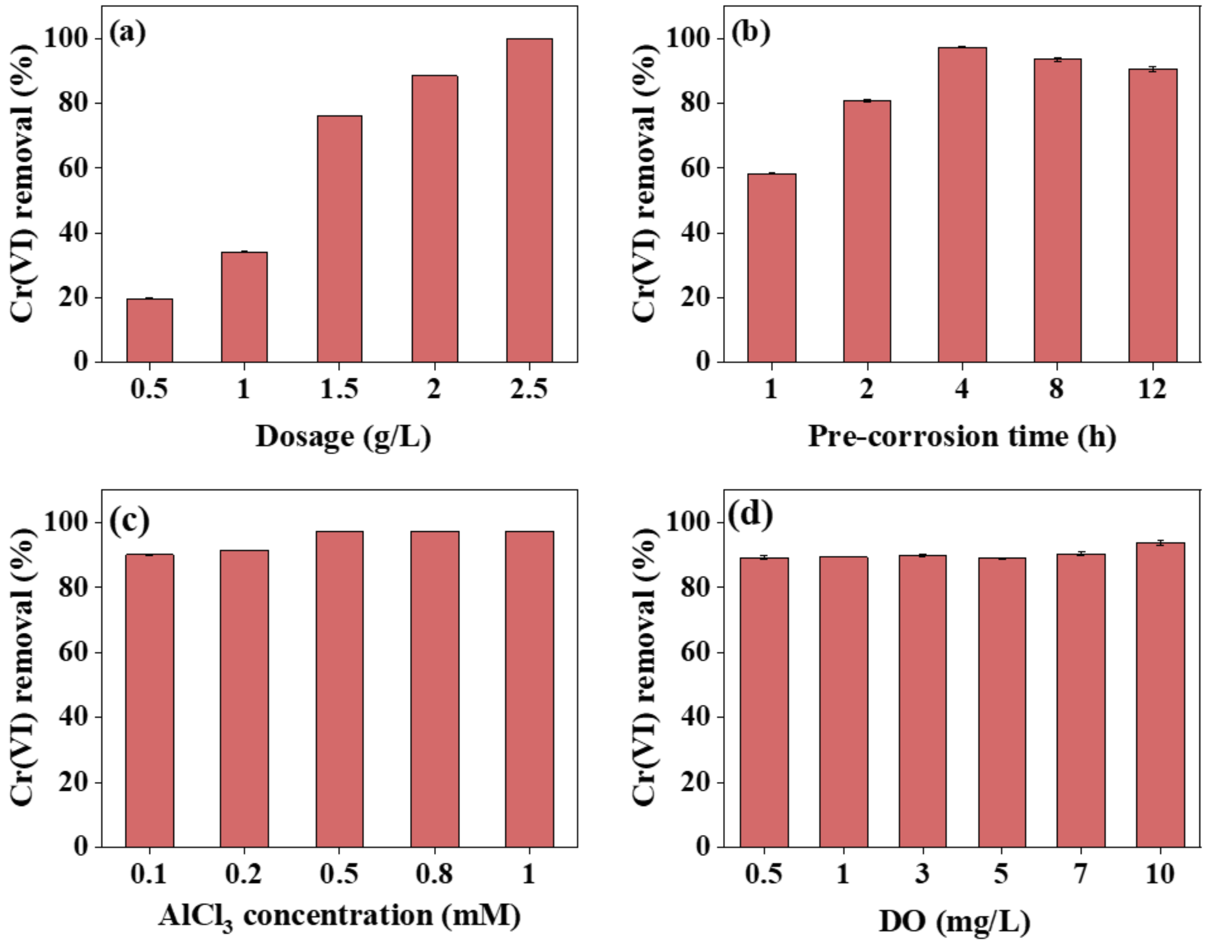

Figure S2. Effect of ZVI dosage (a), pre-corrosion time (b), initial $AlCl_3$ concentration (c) and DO concentration (d) on ZVI pretreatment for Cr(VI) removal. Experimental conditions: (a) ZVI dosage = 0.5-2.5 g/L, $AlCl_3$ = 0.5 mM, pre-corrosion time = 4 h, initial DO = 8.2-8.8mg/L; (b) ZVI dosage = 2.5 g/L, pre-corrosion time = 1-12 h, $AlCl_3$ = 0.5 mM, initial DO = 8.2-8.8mg/L; (c) ZVI dosage = 2.5 g/L, pre-corrosion time = 4 h, $AlCl_3$ = 0.1-1 mM, initial DO = 8.2-8.8mg/L; (d) ZVI dosage = 2.5 g/L, pre-corrosion time = 4 h, $AlCl_3$ = 0.5 mM, initial DO = 0.5-10 mg/L. All experiments were conducted with Cr(VI) = 10 mg/L, pH = 5.0 ± 0.1, T = 25 ^{o}C, reaction time = 3 h.

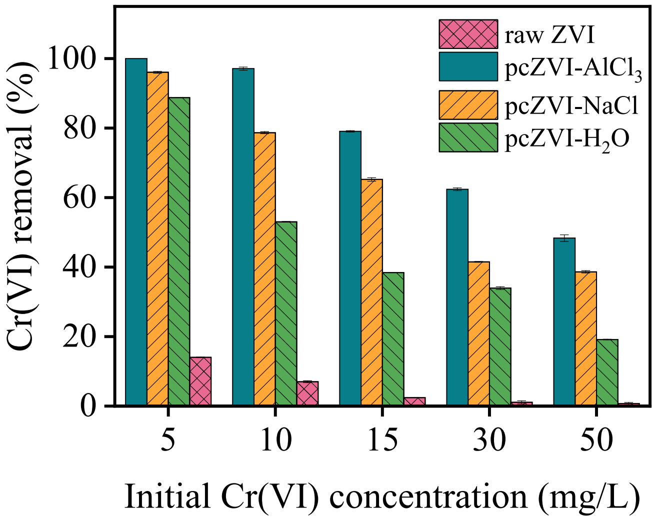

Figure S3. The effect of initial Cr(VI) concentration on Cr(VI) removal efficiency by raw ZVI and after $AlCl_3$ solution, NaCl solution and DI water pretreatment. Test conditions: Cr(VI) = 5-50 mg/L, initial pH = 5.0 ± 0.1, T = 25 °C.

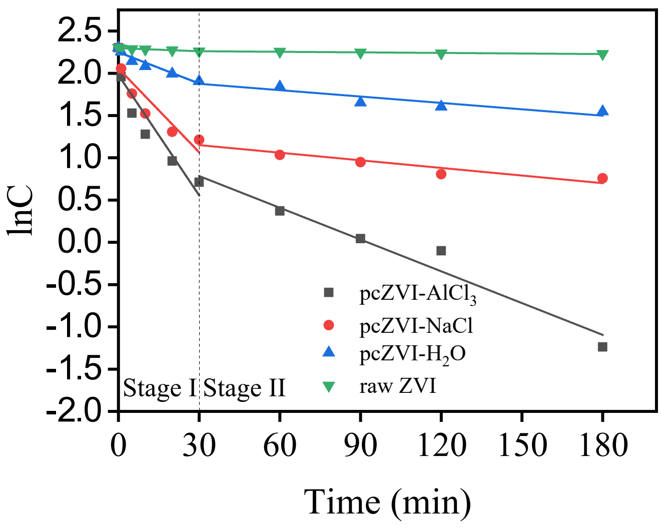

Figure S4. The first-order kinetic model fitting of Cr(VI) removal raw ZVI and after $AlCl_3$ solution, NaCl solution and DI water pretreatment.

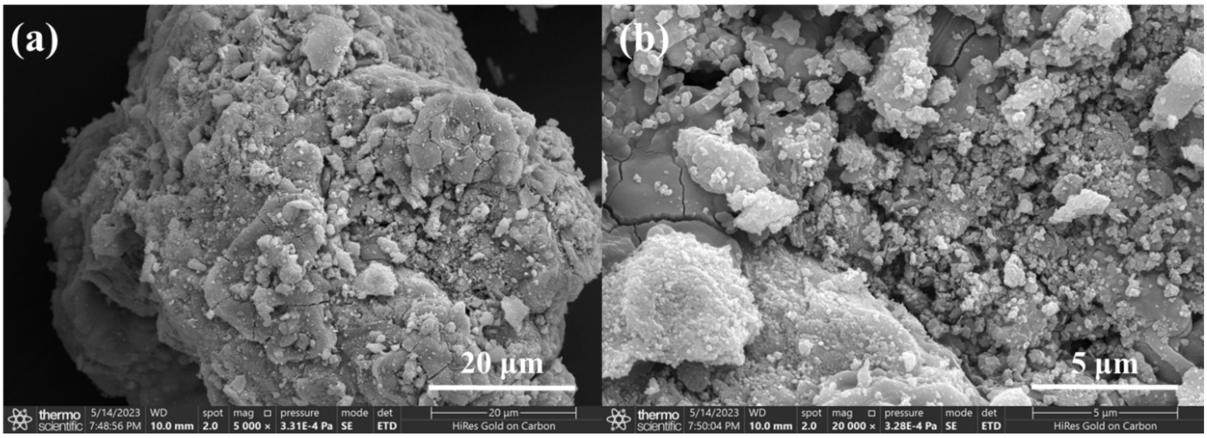

Figure S5. SEM images of the pcZVI-$AlCl_3$ surface with corrosion products peeled off via sonication. (a) 5000×, (b) 20000×.

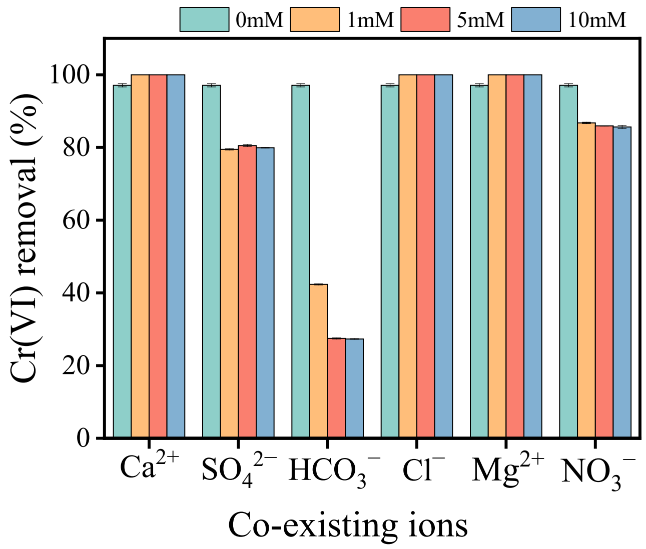

Figure S6. Effect of co-existing cations and anions on Cr(VI) removal by pcZVI-$AlCl_3$. Pretreatment conditions: $AlCl_3$ = 0.5 mM, pre-condition time = 4 h, ZVI = 2.5 g/L. Test conditions: initial pH = 5.0 ± 0.1, Cr(VI) = 10 mg/L, T = 25 ºC, co-existing ions = 0-10 mM.

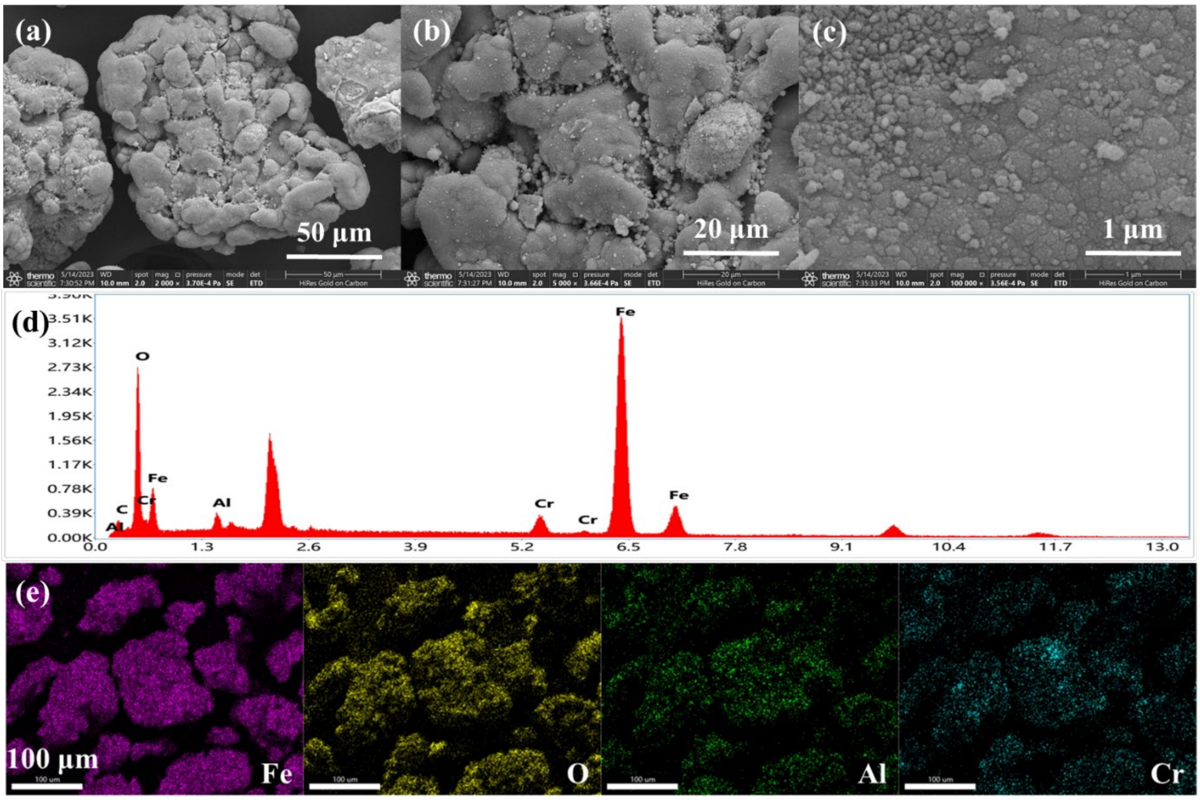

Figure S7. SEM images ((a)-(c)), EDS (d) and Fe, O, Al and Cr elemental mappings (e) of pcZVI-$AlCl_3$ after Cr(VI) removal.

Table S1 The fitting parameters of ^{57}Fe Mössbauer spectra.

Component	Proportion (%)	Amplitude	Isomer shift (mm/s)	Quadrupole Splitting (mm/s)	Magnetic field (T)
Fe^0	49.22%	450.79618	-0.00349	/	47.84306
Fe_3O_4	34.03%	905.53628	0.34938	/	33.04069
FeOOH	16.76%	1332.14598	0.29343	0.82	/

Table S2 Relative contents of Cr 2p in different chemical states of corrosion products in pcZVI after Cr(VI) removal.

Chemical states	Binding energy (eV)	Atomic percent (%)
Cr(III) $2p_{3/2}$	577.04	49.49
Cr(III) $2p_{1/2}$	586.98	32.04
Cr(VI) $2p_{3/2}$	579.00	15.73
Cr(VI) $2p_{1/2}$	589.58	2.74

Table S3 Relative contents of Fe 2p in different chemical states of corrosion products in pcZVI before and after Cr(VI) removal.

	Chemical states	Binding energy (eV)	Atomic percent (%)
Before reaction	Fe(II) $2p_{3/2}$	710.90	34.97
	Fe(II) $2p_{1/2}$	724.30	30.30
	Fe(III) $2p_{3/2}$	713.50	26.02
	Fe(III) $2p_{1/2}$	726.80	8.71
After Cr(VI) removal	Fe(II) $2p_{3/2}$	711.00	37.37
	Fe(II) $2p_{1/2}$	724.38	21.20
	Fe(III) $2p_{3/2}$	713.25	25.13
	Fe(III) $2p_{1/2}$	726.86	16.30

Table S4 Relative contents of O 1s in different chemical states of corrosion products in pcZVI before and after Cr(VI) removal.

	Chemical states	Binding energy (eV)	Atomic percent (%)
Before reaction	OH^-	531.40	55.61
	O^{2-}	530.00	26.50
	H_2O	532.60	17.89
After Cr(VI) removal	OH^-	531.47	45.10
	O^{2-}	530.00	41.70
	H_2O	532.64	13.20

References

1. Rancourt, D.G.; Ping, J.Y. Voigt-based methods for arbitrary-shape static hyperfine parameter distributions in Mössbauer spectroscopy. *Nuclear Instruments and Methods in Physics Research Section B: Beam Interactions with Materials and Atoms* **1991**, *58*, 85-97.
2. Wan, J.; Wu, B.; Lo, I.M.C. Development of Fe^0/Fe_3O_4 composites with tunable properties facilitated by Fe^{2+} for phosphate removal from river water. *Chem. Eng. J.* **2020**, *388*, 124242.
